# Cloning and expression analysis of the gastric carcinoma-related gene, ELCOX3

**DOI:** 10.3892/ol.2013.1595

**Published:** 2013-09-27

**Authors:** RUNLIANG GAN, XIAOMIN LIU, YADONG ZHOU, YERU TAN, HONGGUANG LIU, GUOQING LI, YUNLIAN TANG, HAILONG XIE

**Affiliations:** Cancer Research Institute, University of South China, Hengyang, Hunan 421001, P.R. China

**Keywords:** gastric carcinoma, gene, cytochrome *c* oxidase, ELCOX3, MDSCBC11

## Abstract

Gastric cancer is a pathological process of an accumulation of multigene and multistage mutations. A new gene segment, MDSCBC11, has been previously obtained using a gene chip and is negatively associated with gastric cancer. The present study aimed to clone the full cDNA sequence of the MDSCBC11 segment and to detect its expression in gastric carcinomas and normal gastric mucosa. Multiple-tissue northern blots revealed that the new MDSCBC11-represented gene was expressed as two transcripts that were 0.8 kb and 1.5 kb in size. The cDNA sequence of the smaller transcript was 822 bp, created by 5′ rapid amplification of cDNA ends (RACE) and 3′ RACE methods. A bioinformatics analysis indicated that the deduced amino acid sequence of MDSCBC11 had a 99% homology with the cytochrome *c* oxidase III (COX3) gene in the mitochondria. A total of 46 cases of gastric carcinomas, adjacent gastric mucosa and normal gastric mucosa were individually collected, and the mRNA expression of the ELCOX3 gene was detected by RT-PCR. ELCOX3 mRNA was expressed in all 46 cases of the normal gastric mucosa. The expression levels of ELCOX3 mRNA in the gastric carcinomas were lower compared with that of the adjacent and normal gastric mucosa (P<0.05), with the percent of downregulation at 23.91% (11/46 cases). The downregulation of ELCOX3 gene expression was associated with the development of human gastric carcinomas.

## Introduction

The etiology and pathogenesis of gastric cancer remains enigmatic. Cloning and functional studies of gastric cancer-related genes are important for revealing the gene changes and molecular mechanisms in gastric cancer. As in other malignancies, the development of gastric cancer is a pathological process of an accumulation of multigene, multistage mutations (1–3). A high frequency of loss of heterozygosity (LOH) has been reported on chromosome 1p35–36 for a variety of tumors (4,5), indicating that tumor-related genes exist that have not been cloned in this chromosomal region. Igarashi *et al* identified a high frequency LOH in the chromosome 1p35–36 region in gastric cancer (6). The downregulation of a newly identified gene of the expressed sequence tag segment, MDSCBC11, in the chromosome 1p35–36 region was detected in our previous investigation using cDNA microarray analysis (7). In the present study, the MDSCBC11-represented gene was cloned and its full cDNA sequence was obtained. Gene expression was investigated in gastric carcinomas, adjacent gastric mucosa and normal gastric mucosa.

## Materials and methods

### Northern blotting

Total RNA was extracted from normal human liver tissue and the primer was designed according to the sequence of MDSCBC11. RT-PCR was performed using isotope ^32^P-labeled dCTP as the substrate as follows: 30 cycles of 95°C for 5 min, 94°C for 35 sec, 56°C for 35 sec and 72°C for 35 sec, and a final extension at 72°C for 10 min. The PCR product, which was used as the probe in the following study, was sequenced to confirm that it was consistent with the sequence of MDSCBC11. A northern blot was performed using multiple-tissue northern blots (MTN; Cat. no. 636803; Clontech Inc., Mountain View, CA, USA) with the purified probe.

### Rapid amplification of cDNA ends (RACE) experiment to obtain the full-length cDNA of the MDSCBC11-represented gene

Total RNA was extracted from normal fetal liver tissue, and cDNA was obtained by a two-step RT-PCR. Following this, 5′ RACE and 3′ RACE procedures were performed following the instructions of the SMART™ RACE cDNA Amplification kit (Cat. no. 634914; Clontech Inc.). The primers and amplification conditions are shown in Table I. The PCR products were sequenced. According to sequencing results, the overlapped sequences of the 5′-RACE and 3′-RACE cDNA fragments were removed and the full-length cDNA was obtained.

### Bioinformatics analysis

The bioinformatics-associated software and website (http://www.ncbi.nlm.nih.gov/mapview/) were adopted to predict and analyze the structure and homology of the MDSCBC11-represented gene.

### Detection of ELCOX3 expression in gastric cancer

Specimens from 46 patients with gastric cancer who had not been administered radiotherapy, chemotherapy or other anti-tumor treatments were collected at the First Affiliated Hospital of the University of South China (Hengyang, Hunan, China). The study was approved by the ethics committee of University of South China (Hengyang, China). Written informed consent was obtained from the patients. Three sections consisting of gastric carcinoma, adjacent gastric mucosa at 1–2 cm from the cancer-foci edge and normal gastric mucosa from the cancer-foci edge (>5 cm) were isolated from each specimen within 30 min of being excised from the body. Two to three pieces (~100 mg) for each section were prepared and the tissue pieces were individually transferred to Eppendorf microcentrifuge tubes for liquid nitrogen cryopreservation subsequent to being rinsed with aseptic 0.1% diethypyrocarbonate water. Among the 46 cases, 26 were male and 20 were female, with an age range of 26–77 years (mean, 56 years). There were 11 cases of gastric fundus or cardia cancer, 17 of gastric body cancer and 18 of gastric antrum cancer. There were 19 cases of well- or moderately-differentiated adenocarcinoma, including mucinous adenocarcinoma, and 27 cases of poorly-differentiated adenocarcinoma. Of the total cases, 18 were Borrmann type I+II and 28 cases were type III+IV. There were 24 cases of TNM stages I+II and 22 cases of stages III+IV. Lymph node metastasis was observed in 30 cases and was absent in 16. The clinical data for all the cases were available and all specimens were confirmed by histopathology.

RNA was extracted according to the manufacturer's instructions for the EZNA Total RNA kit (Omega Corp., Guangzhou, China) A two-step RT-PCR procedure was performed and primers were designed according to the sequence of the target gene and the reference glyceraldehyde 3-phosphate dehydrogenase (GAPDH) gene, which were synthesized by Shanghai Invitrogen Biotechnology Co., Ltd. (Shanghai, China). The primer sequences and amplified fragment lengths were as follows: ELCOX3 forward, 5′-CGCGATGTAACACGAGAAAG-3′ and reverse, 5′-TATTAGTTGGCGGATGAAGC-3′ (PCR product size, 500 bp); and reference GAPDH gene forward, 5′-GTCAGTGGTGGACCTGACCT-3′ and reverse, 5′-TGAGGAGGGGAGATTCAGTG-3′ (PCR product size, 400 bp).

### Statistical analysis

All data are expressed as mean ± SD. Data were analyzed using one-way ANOVA, t-test and Spearman's correlation coefficient. P<0.05 was considered to indicate a statistically significant difference.

## Results

### Size and tissue distribution of MDSCBC11-represented gene transcript

**Figure 1:**
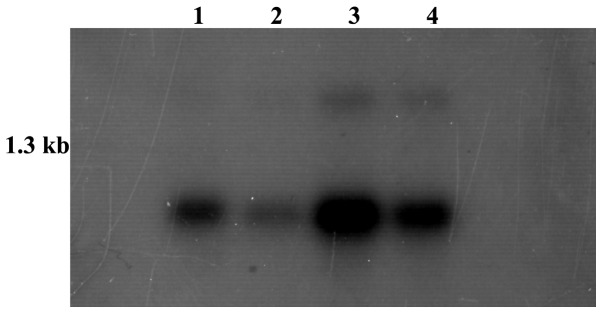
Northern blot analysis of MDSCBC11 in four human fetal tissues. Lane 1, brain; 2, lung; 3, kidney; and 4, liver. The bands in the kidney and liver were ~0.8 kb and 1.5 kb in size, respectively.

A northern blot of α-^32^P-dCTP-labeled MDSCBC11 cDNA with MTN (Cat. no. 636803) revealed that the MDSCBC11-represented gene was expressed in the liver, brain, kidney and lungs. The kidney exhibited the highest expression level and the lung exhibited the lowest expression level. There were two transcripts in the kidney and liver, with a small transcript of 0.8 kb and a larger transcript of 1.5 kb (Fig. 1).

### Cloning of the full-length cDNA of the small transcript (0.8 kb)

**Figure 2:**
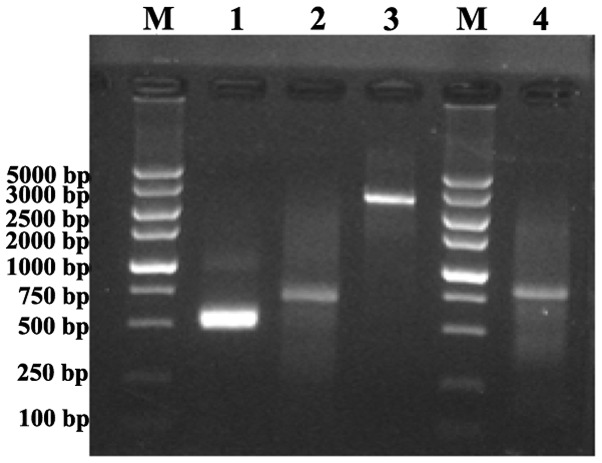
DNA detection by agarose gel electrophoresis. M, DL5000; lane 1, 3′-RACE; lanes 2 and 4, 5′-RACE; lane 3, 3′-RACE control. RACE, rapid amplification of cDNA ends.

The distribution of the 5′ and 3′ end sequences were acquired by RACE using RNA that was extracted from normal embryonic liver tissue by the SMART RACE cDNA Amplification kit (Cat. no. 634914). An agarose gel electrophoretogram revealed that the 5′-RACE product was ~700 bp in size and the 3′-RACE product was ~500 bp in size (Fig. 2). The PCR products were sent to Takara (Kyoto, Japan) for sequencing. According to the sequencing results, the overlapped sequences of 5′-RACE and 3′-RACE cDNA fragments were removed and a full-length cDNA of 822 bp was obtained.

**Figure 3:**
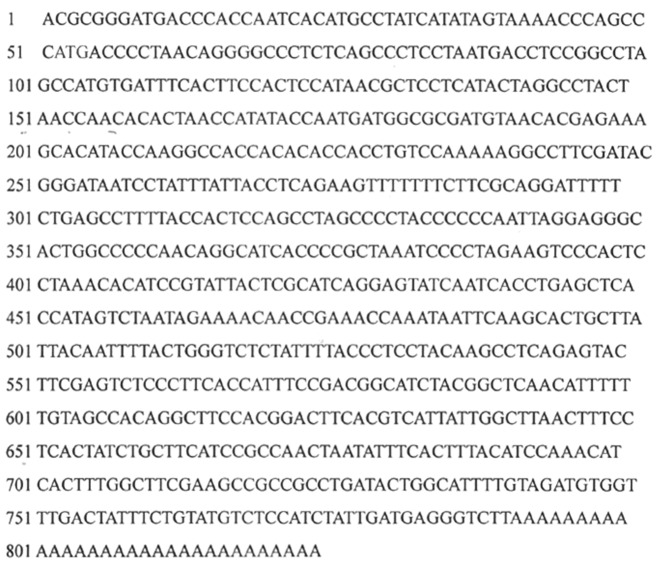
Full-length cDNA of the small transcript sequence.

The full-length cDNA of the small transcript sequence is shown in Fig. 3.

### Bioinformatics analysis of ELCOX3

The results of the BLAST analysis (blast.ncbi.nlm.nih.gov/Blast.cgi) for the MDSCBC11 cDNA indicated that the cDNA of the MDSCBC11 small transcript had 99% homology with the human cytochrome *c* oxidase subunit III (COX3) gene in the mitochondria and was therefore named ELCOX. The ELCOX3 and COX3 genes were blasted and it was observed that the cDNA sequence of COX3 at the 635th C changed to the T of ELCOX3, which resulted in an amino acid codon change from UCA to UUA and an amino acid change at residue 212 from serine to leucine. The results of the chromosome location analysis demonstrated that the ELCOX3 gene was located inside the mitochondria.

Using the open reading frame (ORF) finder server of NCBI (http://www.ncbi.nlm.nih.gov/gorf/gorf.html), the sequence had a complete ORF, which encoded 261 amino acids from the 8th to 793rd bases. The results of the analysis by ExPASy and NCBI BLAST indicated that the ELCOX3 encoded protein had 99% homology with the human COX3 gene and that no CpG islands or introns were detected in the 5′ untranslated regions. The encoded protein was a weak acidic protein with a isoelectric point of 6.78 and a molecular weight (MW) of 29.97 kDa. The domain prediction results revealed that the ELCOX3 encoded protein was a type of COX, polychain transmembrane protein and telomerase, which exists in eukaryotes and the majority of bacteria.

### Expression level of ELCOX3 in gastric cancer

**Figure 4:**
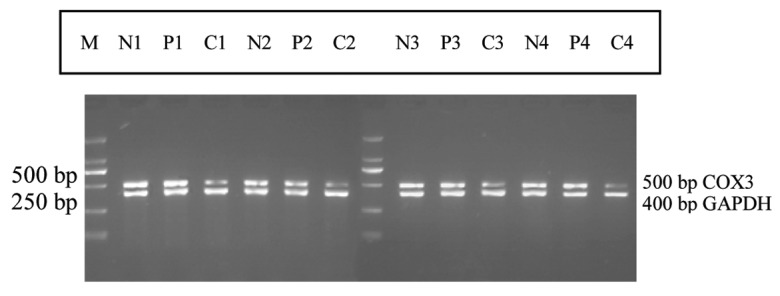
Electrophoresis results of ELCOX3 mRNA expression in gastric cancer. M, DSTM2000; N1–4, normal gastric mucosa; P1–4, adjacent gastric mucosa; C1–4, gastric carcinomas. The ELCOX3 gene expression levels were low in cases 2, 3 (poorly-differentiated adenocarcinoma) and 4 (well-differentiated adenocarcinoma). There was no significant difference between the gastric carcinomas, normal gastric mucosa and adjacent gastric mucosa for case 1 (signet ring cell carcinoma).

From the RT-PCR detection results, the size of the ELCOX3 gene product in gastric mucosa was recorded as 500 bp, while the internal reference of the GAPDH gene product was 400 bp (Fig. 4). The expression level of ELCOX3 in the gastric carcinoma samples was lower than in the adjacent gastric mucosa and normal gastric mucosa samples, with a downregulation of 23.91% (11/46 cases). The optical density ratio analysis revealed that the relative expression values of ELCOX3 mRNA in the gastric carcinomas, adjacent gastric mucosa and normal gastric mucosa were 0.7012±0.1920, 1.1128±0.1605 and 1.1356±0.1537, respectively. The expression level in the gastric carcinomas was significantly lower than that in the corresponding normal gastric mucosa (P=0.016; P<0.05), while there was no significant difference between the corresponding adjacent gastric mucosa and the normal gastric mucosa (P=0.812; P>0.05).

The analysis of the correlation between the clinicopathological parameters of the gastric cancer cases and ELCOX3 expression in the gastric carcinomas demonstrated that the expression of ELCOX3 mRNA in the gastric carcinomas was not correlated with gender, age, tumor size, Borrman classification, differentiation degree, invasion depth or TNM stage (P>0.05). No significant correlation was identified between the downregulation of ELCOX3 mRNA in primary gastric carcinoma and lymph node metastasis (Spearman's correlation coefficient, r=0.088; P=0.559; P>0.05).

## Discussion

Gastric cancer is the result of the interaction of genetic, environmental and other factors, involving changes in the expression and regulation of a large number of genes. In the present study, MDSCBC11 was selected from the differentially-expressed genes that are associated with gastric cancer by cDNA microarray (7) to perform RACE, and 5′ and 3′ end products were acquired. A full-length cDNA of 822 bp was obtained. This gene had no intron and its ORF was located at the 8th to 793rd bases, which encoded 261 amino acids and had a MW of 29.97 kDa. BLAST analysis indicated that ELCOX3 had 99% homology with the COX3 gene. MTN revealed that the MDSCBC11-represented gene had two transcripts, a small transcript of 0.8 kb and a larger transcript of 1.5 kb. The full-length cDNA of the 0.8 kb transcript was obtained in the present study and the 1.5 kb transcript, which may be a new gene that has not been cloned, remains to be investigated.

The animal mitochondrial genome (mtDNA) contains 13 protein genes, including the three subunits of COX, subunits I, II and III (COX1, 2 and 3, respectively), and Cytb, ATP6 and ATP8. These genes are significant components for the inner mitochondrial membrane respiratory chain. The homology of COX1, 2 and 3 is ~80%. Therefore, the cloning and analysis of these genes remains an effective way to investigate the phylogeny and characterization of distant relatives (8,9). Subunits I, II and III of COX are encoded in the mitochondrial genome of eukaryotes and are evolutionarily conserved from bacteria to humans (10). These three subunits constitute the catalytic core of mitochondrial oxidase, as well as the catalytic core of all bacteria aa3 type COX (11–14). Subunit III is more conservative than subunits I and II and cannot transfer electrons directly since it contains no metal centers, but is able to pump protons through cytochrome oxidase. The effect of cytochrome oxidase decreases when the level of subunit III is reduced. However, the specific mechanism is unclear (15–17). In the present study, the stop codon of human ELCOX3 was shown to be T, as in *Anopheles quadrimaculatus*, *Anopheles gambiae* and *Penaeus monodon* (tiger prawns), and a polyA tail is required to be added to codon T as the final stop codon (18–20). There were certain differences between the human COX3 and ELCOX3 sequences. With the exception of codons 1–7, there was one base change; the 635th C changed to a T. The ELCOX3-represented protein was 99% homologous with the human COX3 protein, with only the 212th serine changed to leucine.

mt-COX is a rate-limiting enzyme in cell respiration chain transmission. mt-COX cooperates with cytochrome *c* and plays a significant role in cell mitochondria apoptosis. mt-COX-encoded gene mutations or expression changes may induce biological characteristics and functional changes in its corresponding protein, which thus makes the cell abnormal. mt-COX gene mutation is associated with the development of tumors (21–25), and COX plays a specific role in tumor development, mainly through an increase in reactive oxygen species in mitochondria oxidative phosphorylation.

Semi-quantitative RT-PCR introduced an internal reference as a contrast. The scanning density ratio of the target gene and internal reference gene as the relative expression of the target gene may not only confirm the integrity of RNA extraction and the success of RT-PCR, but also provides the target gene with a quantitative criteria (26,27). In the present study, ELCOX3 mRNA expression in gastric carcinomas, corresponding adjacent gastric mucosa and distal normal gastric mucosa was detected by RT-PCR. The results demonstrated that the expression level in the gastric carcinoma samples was significantly lower than that in the corresponding normal gastric mucosa (P<0.05), while there was no significant difference between the adjacent gastric mucosa and the corresponding normal gastric mucosa (P>0.05). Compared with the corresponding normal gastric mucosa, the expression levels of ELCOX3 mRNA in the gastric carcinomas were downregulated at a rate of 23.91% (11/46 cases). The down regulation of the ELCOX3 mRNA was not correlated with lymph node metastasis. Therefore, ELCOX3 mRNA downregulation may be an early event during the course of gastric cancer and may be associated with the pathogenesis of human gastric cancer.

In summary, the full cDNA sequence of the small transcript of the MDSCBC11-represented gene was identified to be 822 bp in size and was named ELCOX3 due to the homology with the COX3 gene in the human mitochondria. The downregulation of ELCOX3 gene expression was shown to be associated with the development of human gastric carcinomas.

## Figures and Tables

**Table I:** Primer sequences and amplification conditions of the MDSCBC11 segment and RACE.

Primer	Sequence (5′-3′)	Annealing temperature, °C	Product size, bp
MDSCBC11			
F	GCGATGTAACACGAGAAAG	55	392
R	GGAAATGGTGAAGGGAGAC		
GAPDH			
F	AACTGTGGCGTGATGGCCGC	58	500
R	GCAGGGACTCCCCAGCAGTG		
RACE			
F	GCACATACCAAGGCCACCACACA	57	
R	CAGGCATCACCCCGCTAAATCCC		

RACE, rapid amplification of cDNA ends; F, forward; R, reverse; GAPDH, glyceraldehyde 3-phosphate dehydrogenase.

## Abbreviations

COX3: cytochrome *c* oxidase III
